# Starting a new residency program: a step-by-step guide for institutions, hospitals, and program directors

**DOI:** 10.3402/meo.v21.32271

**Published:** 2016-08-08

**Authors:** Michelle Barajaz, Teri Turner

**Affiliations:** 1 Department of Pediatrics, Baylor College of Medicine, The Children’s Hospital of San Antonio, San Antonio, TX, USA; 2 The Department of Pediatrics, Center for Research, Innovation, and Scholarship in Medical Education, Baylor College of Medicine, Houston, TX, USA

**Keywords:** graduate medical education, funding, physician shortage

## Abstract

Although our country faces a looming shortage of doctors, constraints of space, funding, and patient volume in many existing residency programs limit training opportunities for medical graduates. New residency programs need to be created for the expansion of graduate medical education training positions. Partnerships between existing academic institutions and community hospitals with a need for physicians can be a very successful means toward this end. Baylor College of Medicine and The Children's Hospital of San Antonio were affiliated in 2012, and subsequently, we developed and received accreditation for a new categorical pediatric residency program at that site in 2014. We share below a step-by-step guide through the process that includes building of the infrastructure, educational development, accreditation, marketing, and recruitment. It is our hope that the description of this process will help others to spur growth in graduate medical training positions.

In March 2014, the Association of American Medical Colleges (AAMC) Center for Workforce Studies published its report on the 2013 Medical Student Enrollment Survey (1). The results reveal that by 2018, first-year medical school enrollment in the United States is expected to reach a near 30% increase relative to enrollment in 2002. However, 91% of US medial school deans expressed a moderate or major concern about enrollment growth outpacing growth in graduate medical education (GME) on a national level. As of 2011, 19 states already had undergraduate medical education (UME) enrollment that exceeded GME positions in that state.

In addition, a new AAMC report on physician supply and demand projections predicts an estimated shortage anywhere between 46,100 and 90,400 doctors in the United States by 2025. This report calls for an increase in 3,000 Medicare-funded GME positions annually between 2017 and 2021 as one solution to meet these demands (2).

In many institutions, growth of existing programs is limited by patient volume, educational space, and other constraints. In addition, outdated federal funding caps make growth in existing GME programs a non-viable financial option for some hospitals. New programs are not limited by these funding caps, and thus this may be the quickest method for GME expansion. Some states are already increasing funding to help spur this growth. In Texas, the state legislature recently awarded more than $80 million to fund new and expanded program grants, which influenced the development of over 450 new residency positions in a state that already faces an imbalance between UME and GME positions and has several new medical schools in development.

As governments, academic institutions, and independent hospital systems work to meet this increasing physician demand, many directors of new programs will be asking how to best develop their residencies. Very little has been published on this topic. Building a new program can be an exciting opportunity – full of possibility and with options to design an educational experience which truly fits within current and future directions in GME in a novel, nimble environment. The process is not without its challenges, however. These tips are based on personal experience of developing a new pediatric residency program accredited by the ACGME (The Accreditation Council for Graduate Medical Education) at a community hospital for an established US academic institutional sponsor. What follows is a ‘nuts and bolts’ guide to navigating this process which we hope will help others in some way. In reality, many of these steps occur concurrently in a joint effort by stakeholders both at the training sites and the institution (Table 1).

**Table 1:** Starting a new residency program

Recommended timeline	Building the infrastructure	Educational development	Program accreditation	Marketing and recruitment
6–12 months	Institutional affiliation and training site			
	Budget/funding Timeline			
3–4 months		Review and apply ACGME requirements		
		Develop your educational vision		
4–6 months		Curriculum and program design	Complete the application	
2–3 months		Faculty development	Prepare for site visit	
3–6 months			Await accreditation decision	Strategic marketing
8 months (July–February)				Active recruitment
4 Months (March–June)				The beginning: match and orientation

## Building the infrastructure

### Establish an institutional affiliation or sponsorship and locate a primary training site

Every residency must first have an accredited institution as its sponsor. The startup process can be made much more expeditious when a relationship is secured with an established academic institutional sponsor. There can be benefits to both parties. More and more institutions are successfully using this as an expansion tactic, developing programs in other cities and even outside of their own states or countries. Collaboration with community hospital partners in remote sites can not only build tertiary referral bases but also help meet medical needs in underserved areas. Existing institutional sponsors have expertise which places them in the best position to develop new residency programs for community hospitals, and the reputation of the institution can lend credence to the venture. The collaboration also strengthens the credibility of a fledgling program among applicants, and an academic partner with a medical school may be a helpful recruitment stream for potential candidates. The addition of educational programs often increases the stature of the hospital within the community and ultimately can provide a long-term solution for physician shortages in the area as residents begin to graduate the program.

Next, the primary training site must be selected, taking into consideration the adequacy of facilities, patient volume, and teaching faculty. If ancillary sites are needed to supplement the educational experience, Program Letters of Agreement (PLA) will need to be arranged prior to the first accreditation site visit for each required rotation that takes place there. At the minimum, a program director and coordinator located at the primary site will need to be selected early on in the process of accreditation, with additional supporting staff a strong consideration. The Designated Institutional Official (DIO) will need to be identified within the sponsoring institution and will be the initiator of the application process with the ACGME.

### Develop a budget and secure funding for the program

Funding for resident salaries and benefits, administrative faculty time, recruitment, and other operating costs will need to be arranged. In the United States, some federal funding will go to the primary training site; however, prior to the establishment of a revenue stream, part of the startup burden may need to be shared by the sponsoring institution. Some administrative costs will occur 2 or more years prior to the induction of the first trainees. Given that nearly 53% of residents stay in the states where they are trained, some state legislatures have now begun offering startup and expansion grant funding for new GME positions, particularly in primary care (3, 4). Private grant funding may also be a viable startup funding source. Loan repayment options in underserved areas should also be explored as a potential supplemental benefit for residents. A residency training program can be viewed as a resource for the community and buy in from local governments and philanthropists may improve opportunities for unique forms of sponsorship. This step, along with an assessment of patient volume, will likely determine the appropriate startup size for your program.

### Determine a reasonable timeline for application, accreditation, recruitment, and initiation of your program

This step hinges on ACGME Residency Review Committee (RRC) meeting dates for your specialty. Approximately 1 year is normally needed for preparation of the application and completion of the site visit process. In addition, a minimum of 1 year will be needed after the accreditation for the recruitment and match process, starting in the summer when students begin applying to programs. If the accreditation decision is made after July, strong consideration should be made to putting off the start of the program for another academic year due to difficulty in marketing the program to potential applicants in time for the residency interviews. Because of these intricacies, mapping out a timeline early is crucial.

## Educational development

### Immerse yourself in the ACGME requirements and determine how your program will meet each one

A primary role of the program director is to ensure that the program meets all the ACGME requirements. Reviewing the institutional, common, and specialty-specific program requirements in detail is an important and ongoing task. Typically, the language of the requirements includes ‘must have’ and ‘should have’ recommendations, as well as some requirements which leave a great deal of flexibility in their application. Ensure that all sections of the ACGME website are explored, especially the ‘frequently asked questions’, power point presentations, and notable practices sections, as they often provide clues as to the preferred interpretations of some of these guidelines. In general, the ACGME staff are amenable to answering questions that remain when the website cannot provide the exact answers that are needed. The program directors association for your specialty can be another valuable resource and can provide remarkable insight into the ways that other programs have implemented the guidelines and requirements put forth by the ACGME.

### Develop your educational vision

For true educators at heart, the next two steps are the most invigorating and should be approached as a golden opportunity. Take stock of your resources – unique clinical programs, stellar faculty, facilities, patient population, hospital organizational structure, positioning within the community, etc. What will be your program's unique niche? What educational approaches will best suit your situation? How will you advance education in your field? Few get the chance to consider these questions and develop a program from scratch that perfectly fits every aspect of the existing infrastructure, and without the constraints of having to worry about complete coverage of the hospital or even a particular service by their residents. Carefully consider the ideal balance of service and education in order to structure this from the start. Working in conjunction with institutional, departmental, and hospital administration ensures that the vision becomes unified early and helps to eliminate differences of opinion that will bog down your application process.

### Initiate curriculum and program design

Once you have decided on your educational approach, you must begin planning your curriculum, ensuring that you meet every aspect of the program requirements in your specialty. Not only must you develop the overall plan for clinical rotations but also for didactic teaching, board review, and any other longitudinal curricula you wish to implement. Rotation directors with a strong interest in medical education should be selected and recruited to help plan the program and write competency-based goals and objectives for each clinical rotation. It is important to create an overall educational map, determining how you are teaching and assessing each competency in multiple educational arenas and over time. Much assistance in these tasks can be gained from researching MedEdPORTAL^®^ (www.mededportal.org/) and building on the strong work of other educators who have been willing to share their curricula.

It is important to consider at this step how you will meet requirements for program evaluation, assessing clinical competency, and ensuring the quality and safety of the clinical learning environment. If your institution does not already have them in place, formal policies for duty hours, supervision, moonlighting, etc., will need to be drafted. Planning for research involvement for your residents, counseling support and wellness programs, and integration of resident documentation in the electronic medical record may require additional infrastructure to be developed within your institution, and space for didactic conferences, call rooms, and GME administration will need to be identified. Strong consideration should be given to the use of electronic residency management evaluation systems and a web-based educational platform for more expeditious and documented dissemination of information between program leaders and residents.

### Ensure alignment of faculty/faculty development

At this point, you would be wise to begin selecting and engaging your faculty and bringing them into the vision. We set up regular bimonthly faculty development sessions which started more than 18 months prior to the start of our program to begin informing, motivating, and developing educational skills in our faculty, and to align those who had come from many different institutions. It can be a huge cultural shift to introduce GHE into a primarily non-academic environment, but if you are already at an academic institution, enlisting existing faculty development resources can streamline the process. Business literature, such as Transformational Leadership by Bass and Riggio (5) or Leading Change: Why Transformation Efforts Fail by JP Kotter (6), can provide great inspiration on how to implement large-scale changes in the workplace. Communication, education, and cooperation among the faculty are key.

## Program accreditation

### Complete the application

The tedious process of completing the application(s) for accreditation requires an incredible amount of data and careful attention. The DIO must grant initial access to the electronic application system. Wording and framing are very important to ensure that you are addressing the aspect(s) of the ACGME requirements that a particular question is actually aiming to assess. Seek experienced outside reviewers to take a look at your responses with a critical eye and make sure you are explaining your program in a way that can be easily interpreted by the review committee. As you compile the application, maintain supporting documentation for each answer in preparation for the site visit. This is traditionally done by preparing sets of binders by category: faculty information, curricula, policies, patient data, etc. Once completed, the application will be submitted first to the DIO, and then to the ACGME for review.

### Ensure adequate preparation for the site visit

After review of your application by the ACGME, you will be notified of a date for your site visit. They will be interviewing program leadership, administration, and a selected group of faculty and will assess concordance on all aspects of the application. Each group will need to be prepared to verify the information contained in your application, so appropriate information distribution and education on program requirements is key. We developed binders for our faculty team which detailed the most important details of the overall program design and served as an important reference for the aspects of the program application which pertained to their own clinical areas. A ‘mock site visit’ to let faculty know what they will experience that day can help to ease nerves and ensure that you are adequately prepared. This may be conducted either by someone who has extensive ACGME experience or by someone who has just gone through the accreditation process.

## Marketing and recruitment

### Develop your marketing strategy

As you wait for the accreditation decision, develop your marketing plan. How will you get the word out to students about your program? Establishing a web presence early is extremely important, and this includes not only the development of a vibrant website but also registering early with FRIEDA Online^®^, Electronic Residency Application Service^®^ (ERAS), and the National Residency Matching Program^®^ (NRMP), if those are appropriate for your situation. Utilize faculty contacts at home schools, and consider contacting local and regional student groups in your specialty and offering to speak or send information. Many schools offer residency fairs where your program can be advertised. Clerkship directors in your region who mentor medical students should also be informed of your accreditation and opening as soon as the decision is official. Consider offering an open house of some sort where students can come and learn about your vision. Think strategically about your market niche and what will be the likely biggest selling points for your program, and be prepared to answer every email with an overabundance of information. Remember that each student you come in contact with has many friends not only in their class but in the years below them as well.

### Engage everyone in active recruitment

Recruitment into a new program can be very challenging. In the 2013 National Residency Matching Program applicant survey, more than 60% of applicants cited reputation of the program, quality of residents in the program, work life balance, and house staff morale as important in their ranking decisions, all factors which cannot readily be assessed in a new program. In addition, concrete data such as board passage rates, specialty choices, and fellowships obtained by previous graduates are simply not available. Other concerns, such as lack of upper-level resident mentorship, perceived readiness of hospital and personnel to transition to an academic environment, and a simple fear of a program just not having ‘all the kinks worked out’ may also have a negative impact on a candidate's interest in a new program. There are, however, factors which may be attractive to a candidate in a new program, which could be highlighted during the recruitment process. The ability to work one on one with faculty, especially during the first year, and the ability to help shape and develop a training program could be very appealing to some candidates. Personal attention, a high level of involvement with the program director on the day of the interview, and many opportunities to interact with faculty during their visit can help to reassure applicants about their concerns. Seek to identify candidates with the right type of independent and pioneering spirit which will be required of your first class. Always remember that part of the recruitment process is marketing, as students will take their impressions of your program back to their home institutions even if they do not end up matching with your program. Consider interviewing 15–20 candidates per spot and be open to interviewing not just your top candidates but also others who will ensure your program is marketed within a large geographical spread. Your faculty will need to take the place of residents in the recruitment process and exposure to as many faculty as possible during the interview experience is key, as the residents will be working closely with them and will be reassured by the opportunity to interact. When developing your rank list for the match, it is important to list as many applicants as possible to ensure that your program fills, and equally important to never list anyone that you absolutely would not want in your program.

### Enjoy the beginning

Match day will come and the residents will soon follow. Take time to celebrate your successes and hard work! Reward the faculty for their participation with recognition of their efforts. After your residents are selected, the lengthy onboarding process will begin. It is important to engage hospital staff in this process so that they become comfortable and understand the residents’ new roles. Orientation is an important time – a careful balance between giving the new interns the amount of information they need without overwhelming them with things they will likely forget is crucial, as is having a written or electronic resource for all information given that theywill be able to refer back to in the future. It will be an exciting time for everyone to see all your hard work come to fruition, and taking a few moments to enjoy the momentous beginning of your program will give you the fuel to dive into the process of training your residents with renewed vigor.

## Conclusion

Existing academic institutions have a critical role to play in integrating with and investing in their surrounding communities to address US medical workforce needs. As the GME community enters into a rapid growth phase, it will be in need of data and resources to help in the building of new residency programs. It is important to share lessons learned by our experiences and success at The Children's Hospital of San Antonio in order to enable others to build on what we have already learned. While our experience may not be translatable to the structure of every program, these overarching principles will help guide others through the basic steps of creating a brand new residency program and will hopefully encourage them in the process.
